# MI-MAAP: marker informativeness for multi-ancestry admixed populations

**DOI:** 10.1186/s12859-020-3462-5

**Published:** 2020-04-03

**Authors:** Siqi Chen, Sudhir Ghandikota, Yadu Gautam, Tesfaye B. Mersha

**Affiliations:** 1 Department of Pediatrics, Cincinnati Children’s Hospital Medical Center, University of Cincinnati, 3333 Burnet Avenue, MLC 7037, Cincinnati, OH 45229-3026 USA; 2 Department of Electrical Engineering and Computer Science, University of Cincinnati, Cincinnati, OH 45221 USA

**Keywords:** Aancestry informative markers, AIMs, Lancaster estimator of Independence, LEI, MI-MAAP, Admixed population, Admixture mapping

## Abstract

**Background:**

Admixed populations arise when two or more previously isolated populations interbreed. A powerful approach to addressing the genetic complexity in admixed populations is to infer ancestry. Ancestry inference including the proportion of an individual’s genome coming from each population and its ancestral origin along the chromosome of an admixed population requires the use of ancestry informative markers (AIMs) from reference ancestral populations. AIMs exhibit substantial differences in allele frequency between ancestral populations. Given the huge amount of human genetic variation data available from diverse populations, a computationally feasible and cost-effective approach is becoming increasingly important to extract or filter AIMs with the maximum information content for ancestry inference, admixture mapping, forensic applications, and detecting genomic regions that have been under recent selection.

**Results:**

To address this gap, we present MI-MAAP, an easy-to-use web-based bioinformatics tool designed to prioritize informative markers for multi-ancestry admixed populations by utilizing feature selection methods and multiple genomics resources including 1000 Genomes Project and Human Genome Diversity Project. Specifically, this tool implements a novel allele frequency-based feature selection algorithm, Lancaster Estimator of Independence (LEI), as well as other genotype-based methods such as Principal Component Analysis (PCA), Support Vector Machine (SVM), and Random Forest (RF). We demonstrated that MI-MAAP is a useful tool in prioritizing informative markers and accurately classifying ancestral populations. LEI is an efficient feature selection strategy to retrieve ancestry informative variants with different allele frequency/selection pressure among (or between) ancestries without requiring computationally expensive individual-level genotype data.

**Conclusions:**

MI-MAAP has a user-friendly interface which provides researchers an easy and fast way to filter and identify AIMs. MI-MAAP can be accessed at https://research.cchmc.org/mershalab/MI-MAAP/login/.

## Background

Markers with large differences in allele frequencies between ancestral populations, known as ancestry informative markers (AIMs), provide genetic information useful to infer ancestry [1]. Genetic ancestry inference is an area of considerable interest in disease genetics, population genetics, anthropology and forensics and genetic testing [2]. Identification of such a subset of genetic markers that is sufficiently informative for inferring genetic ancestries but still small enough to reduce the computational cost for resource-limited laboratories is of particular importance. With the rapid development of the field of human genetics, enormous amounts of genetic variation in large data repositories have been generated, such as 1000 Genomes Project, HapMap Project, Human Genome Diversity Project (HGDP) and Exome Aggregation Consortium (ExAC) [3–5]. Due to the growing number of the available genomic data, new computational analytics are required to effectively process massive SNP data and extract informative AIMs for multiple racial ancestries.

The task of identifying informative markers to assign individual genomic regions to correct ancestries can be difficult in admixed populations with multiple ancestral origins [6, 7]. Hence, prioritizing AIMs using various feature selection methods is of paramount significance in studies of population structure and to map risk loci via admixture mapping [8]. One of the most powerful methods for identifying such informative AIMs is to apply feature selection techniques where subsets of relevant SNPs are selected based on the classification model construction [9]. Machine learning techniques such as Support Vector Machine (SVM), Random Forest (RF) and Principal Components Analysis (PCA) can be applied to score and rank informative SNPs by modeling classifiers. Numerous AIM panels from autosomal chromosomes have been published [1–5, 8, 10]. Briefly, these studies have shown that 60–150 informative markers are useful for population clustering into major geographic regions [9, 11, 12]. Previous work to develop AIMs considered binary classification with pairs of sub-populations [12]. There are limited statistical tools to develop informative markers for three-way or multi-way admixed populations including Latino populations [11–13]. It is thus desirable to develop an efficient feature selection method, which not only identifies AIMs to estimate the admixture proportions in samples from multi-way admixed population with high accuracy, but is also computationally feasible for small laboratories.

In this study, we extend the two-way ancestry analysis into multi-way ancestry classification, and present a user-friendly web-based tool called Marker Informativeness for Multi-Ancestry Admixed Populations, MI-MAAP, to facilitate selection of informative SNPs in multi-admixed population using 1000 Genomes Project, Human Genome Diversity Project as well as user-generated data. It adopts a novel likelihood-based feature selection method, Lancaster Estimator of Independence (LEI), which has the ability to efficiently compare multiple ancestral populations using allele frequency information [14]. LEI potentially reduces the computation costs associated with individual-level genotype data and yields cost-efficient AIMs panel for ancestry inferences. The lack of easy-to-use-analytical tools is a substantial barrier for most biologists with limited computer programming background. Exploiting recent advancements in web framework technologies the MI-MAAP was developed to simplify informative marker selection for genetic studies in admixed populations. Users are able to freely access MI-MAAP and retrieve large genomic datasets and extract AIMs on-the-fly through the user-friendly interface. We also further investigated the identified SNPs or their nearby genes, with specific functional information and associated diseases that are known to be prevalent in certain ancestry. Additional information (e.g. gene expression, gene ontology, pathway information, protein information and species orthologs) is made available for users to explore their AIMs known to be prevalent in certain ancestry.

## Implementation

### Measure of marker Informativeness

Lancaster estimator of independence (LEI) is a maximum likelihood-based estimator of ancestry informativeness of markers using allele frequency data under Hardy-Weinberg Equilibrium (HWE). LEI can compute the quantitative score from the marker-population joint distribution and thus account for 3 or more populations simultaneously. Let *m* and *M* be the alternate and reference alleles at a marker and *X* = (*x*_1_ = *mm*, *x*_2_ = *mM*, *x*_3_ = *MM*) be the three genotypes. Let *Y* = (*y*_1_, *y*_2_, …, *y*_*k*_) be the *k* distinct ancestral populations and *f*_*j*_ represents the population reference allele frequency in the *j*^*th*^ population with *c*_*j*_ indicating the number of individuals in the *j*^*th*^ population. Then, under the assumption of HWE in individual populations, the expected genotype counts of genotype *x*_1_, *x*_2_, and *x*_3_ in the *j*^*th*^ population can be expressed as ${\hat{n}}_{1j}={c}_j{\left(1-{f}_j\right)}^2,{\hat{n}}_{2j}=2{c}_j{f}_j\left(1-{f}_j\right),\kern0.5em$ and ${\hat{n}}_{2j}={c}_j{f}_j^2$, respectively. If *n* = *c*_1_ + *c*_2_ + … + *c*_*k*_ is the total sample size, the joint probability distribution of *X* and *Y*, *p*_*ij*_ = *P*(*X* = *x*_*i*_, *Y* = *y*_*j*_) can be estimated as ${\hat{p}}_{ij}=\frac{{\hat{n}}_{ij}}{n}$, *i = 1, 2, 3* and *j = 1, 2, …,k.* Then, the Lancaster estimator of independent (LEI) is defined as –

1 $$ {\hat{\theta}}^2=\kern0.5em {\sum}_{i,j}\frac{{\hat{p}}_{ij}^2}{{\hat{p}}_{\mathrm{i}+}{\hat{p}}_{+j}}-1 $$

where ${\hat{p}}_{i+}=\frac{\sum_j{n}_{ij}}{n}=P\left(X={x}_i\right)$ and ${\hat{p}}_{+j}=\frac{\sum_i{n}_{ij}}{n}=P\left(Y={y}_j\right)$ are the marginal distributions of *X* and *Y*, respectively. ${\hat{\theta}}^2$ is the measure of the magnitude of independence between the two categorical variables *X* and *Y*, with ${\hat{\theta}}^2=0$ if *X* and *Y* are independent and $0\le {\hat{\theta}}^2\le \min \left(3,k\right)-1$ [15] . In particular, for genotype data with biallelic markers, $0\le {\hat{\theta}}^2\le 1$ when the sample constitutes two populations (i.e. *k* = 2) and $0\le {\hat{\theta}}^2\le 2$ when the sample constitutes three or more populations (i.e. *k* ≥ 3).

### Programmatic architecture

MI-MAAP was built on the Django web development framework which can be run on different operating systems. PHP APIs were developed to retrieve SNP data from the local database and Python scripts were written to access them and perform feature selection calculations. Python’s Scikit-Learn library was used to conduct PCA, SVM, and RF. The combination of HTML and CSS was used to construct the user interface. In addition, jQuery Javascript library as well as jQuery plugins were used to build dynamic input forms, add interaction controls to output tables, and assist in some client-side design details. The tool was deployed on Apache httpd web server.

## Results

### Workflow of MI-MAAP

**Fig. 1:**
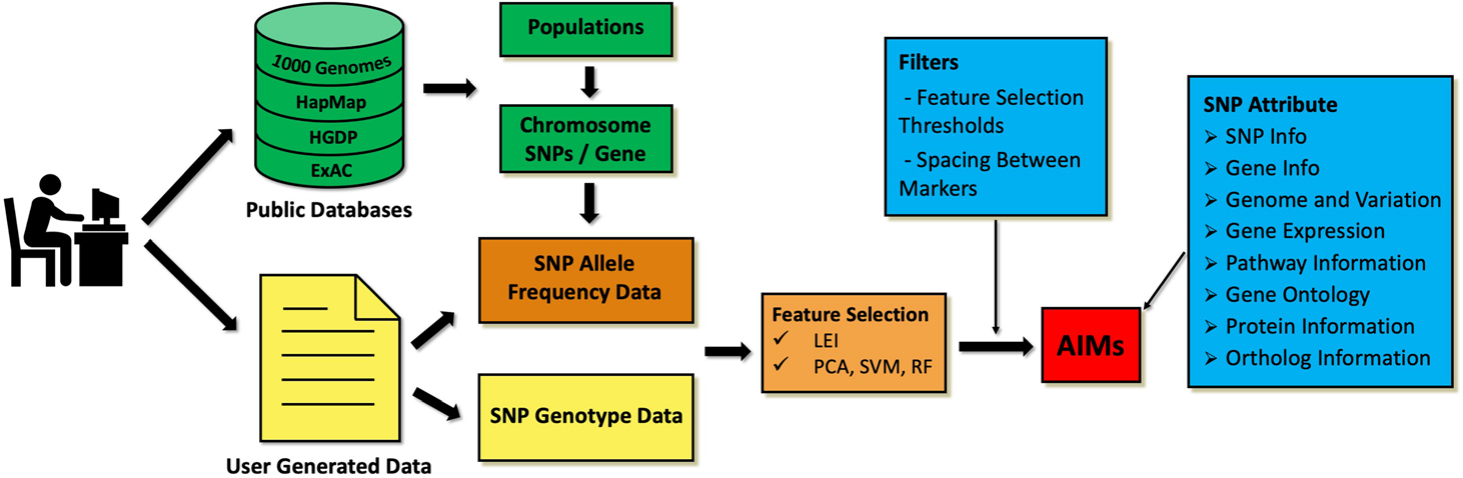
Schematic representation of MI-MAAP workflow. In the flow diagram, once the user enters the information and clicks the submit button, information is mined from specific local databases, and MI-MAAP algorithm computes all comparisons for LEI to output Ancestry Informative Markers (AIMs)

MI-MAAP was designed as a web-based tool for analyzing the marker informativeness in multi-ancestry admixed populations. Its workflow is described in Fig. 1. Users have two types of input options: a) users can input a chromosome, SNP list or a single gene they are interested in from public databases (e.g., 1000 Genomes Project, International Haplotype Map (HapMap), Human Genome Diversity Project (HGDP) and Exome Aggregation Consortium (ExAC) [3–5, 10]; b) users can also upload their own SNP data files that include the population-specific allele frequency or genotype information. When the input is provided as chromosome or SNPs/gene, MI-MAAP first maps chromosomes or genes to their corresponding SNP sets and then uses a local database to retrieve and collect the population-specific SNP allele frequencies from the selected database.

### Databases and populations

**Fig. 2:**
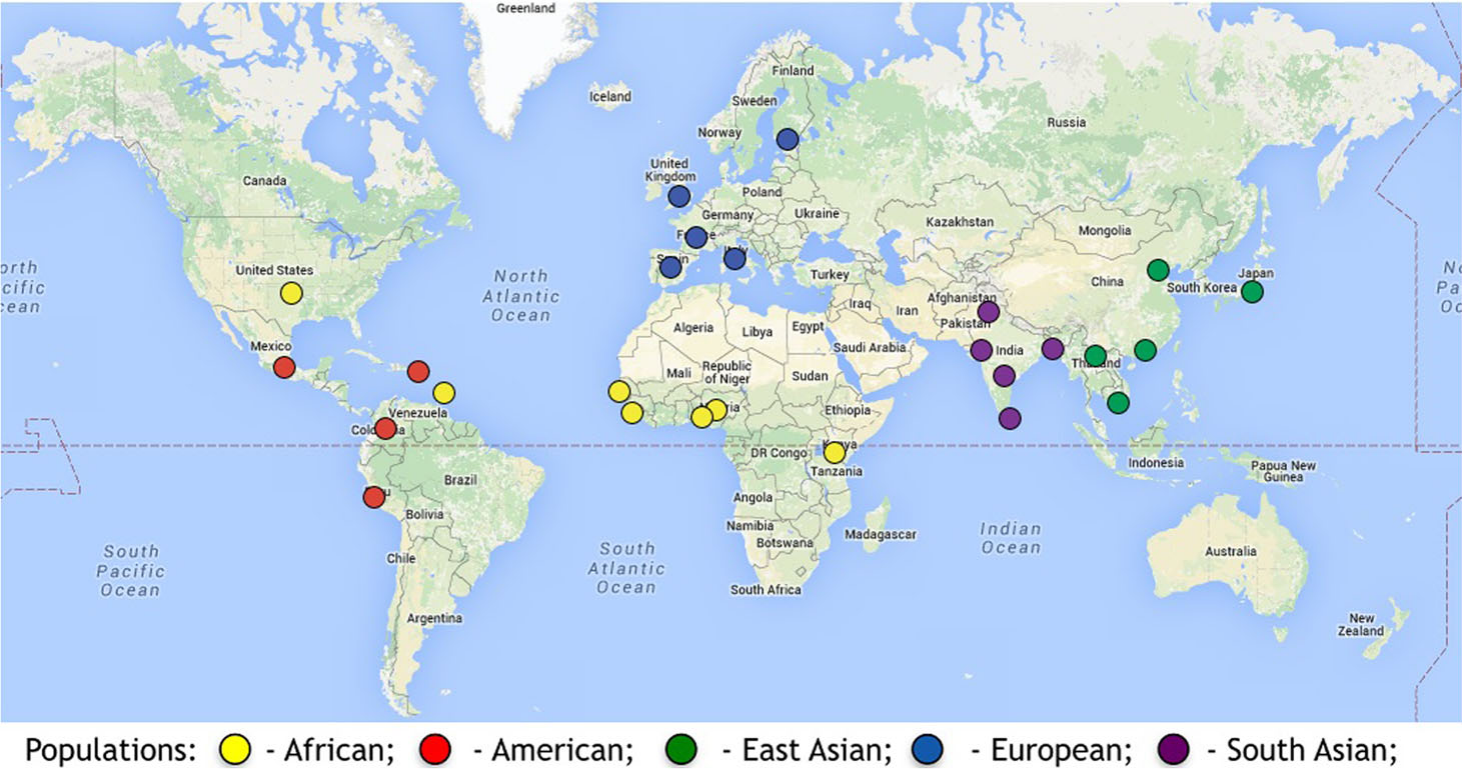
Geographical distribution of 26 populations in the 1000 Genomes Project. The major continental regions are represented in 1000 Genomes Project. Source: The International Genome Sample Resource (IGSR) and 1000 Genomes Project https://www.internationalgenome.org)

The local database stores allele frequency data from four different public data repositories: the 1000 Genomes Project [10], HapMap [16], Human Genome Diversity Project (HGDP) [5] and Exome Aggregation Consortium (ExAC) [17]. Databases utilized and their number of populations and sample sizes with the number of SNPs are presented in Table 1. For example, the default database, 1000 Genomes Project Phase III data, includes a total of 2504 samples from 26 populations (Fig. 2). Reference population with labels, sample sizes that are available from each public database to infer ancestry and web link are provided in Additional file 1: Table S1. The 1000 Genomes Project (*n* = 84.7 million SNPs) across chromosomes are provided in Table 2.

**Table 1:** Reference populations and sample size with the number of SNPs databases accessed by MI-MAAP

Database	Populations	Individuals	SNPs
1000 Genomes	26	2504	84.7 million
HapMap	11	1397	3.2 million
HGDP	53	1043	657,000
ExAC	6	60,706	10 million

**Table 2:** Number of SNPs on each chromosome for 1000 Genomes Phase III dataset

Chr	No. of SNPs	Chr	No. of SNPs	Chr	No. of SNPs
1	6,468,094	9	3,560,687	17	2,329,288
2	7,081,600	10	3,992,219	18	2,267,185
3	5,832,276	11	4,045,628	19	1,832,506
4	5,732,585	12	3,868,428	20	1,812,841
5	5,265,763	13	2,857,916	21	1,105,538
6	5,024,119	14	2,655,067	22	1,103,547
7	4,716,715	15	2,424,689	X	3,468,093
8	4,597,105	16	2,697,949		

### Feature selection methods

After obtaining population-specific SNP data from either the public database or the user uploaded file, feature selection methods will be performed. LEI is an efficient likelihood-based approach which is computed by using the reference allele frequency data to estimate the genotype count data based on the Hardy-Weinberg Equilibrium (HWE) assumption; while PCA, SVM, and RF require the individual-level genotype data. When using summary-level allele frequency data, LEI will be applied. When using user-generated individual-level genotype data, one of the feature selection methods from LEI, PCA, SVM and RF can be performed. If LEI is chosen for genotype data, estimated allele frequencies will be first calculated from the genotype data and then used to compute LEI values. Thresholds for the computation values of the selected feature selection method can be set to filter relevant SNPs. In order to select the most informative SNPs and remove redundant and uninformative SNPs, we implemented filtering criteria including spacing between markers to account the linkage disequilibrium (LD). Removal of redundant and uninformative SNPs reduces the time and genotyping costs while increasing the power for extracting the most informative panels of SNPs. MI-MAAP also provides various ways to export the output, including Excel, CSV and PDF files. The ability of LEI to determine ancestry proportion in multi-ancestry admixed populations using allele frequency data without requiring the individual-level genotype data provides a more efficient alternative to the existing computationally and time-consuming genotype-based analysis for ancestry inference. When other choices are made instead of LEI, we recommend using Random Forest or SVM. In our analyses, the PCA based approach requires a larger set of informative markers to achieve similar performance as of LEI, RF, and SVM to estimate the ancestry proportion of admixed samples. We would also like to point out that the reference panels used for the construction of an informative marker set should be representative of the samples. We suggest developing AIMs from representative populations of interest.

### Attributes to SNPs/genes

In addition to prioritizing AIMs, MI-MAAP allows to retrieve and explore functional annotation information associated with AIMs. These attributes are grouped into eight categories: SNP information (chromosome, alleles, MAF, functional class, Regulome Score, TSS Score, and links to GWAS Catalog, dbGap, Exome variant, Genome variant and so on), gene information (such as gene ID, gene symbol, synonyms, gene description, CpG sites and mapped diseases), genome and variation (links to ENCODE, dbVar, ClinVar and BioGPS), gene expression (such as GEO profiles, GTEx eQTL, Blood eQTL and so on), biological pathways (links to KEGG pathways, Reactome and BioCarta), gene ontology (cellular components, biological process and molecular function), protein (links to UniProt, Protein Atlas, PFAM and SMART), and species orthologs (such as Entrez IDs for chimp, rhesus, mouse, rat, zebrafish, cattle, chicken and dog) [7, 18, 19].

### Components of MI-MAAP

**Fig. 3:**
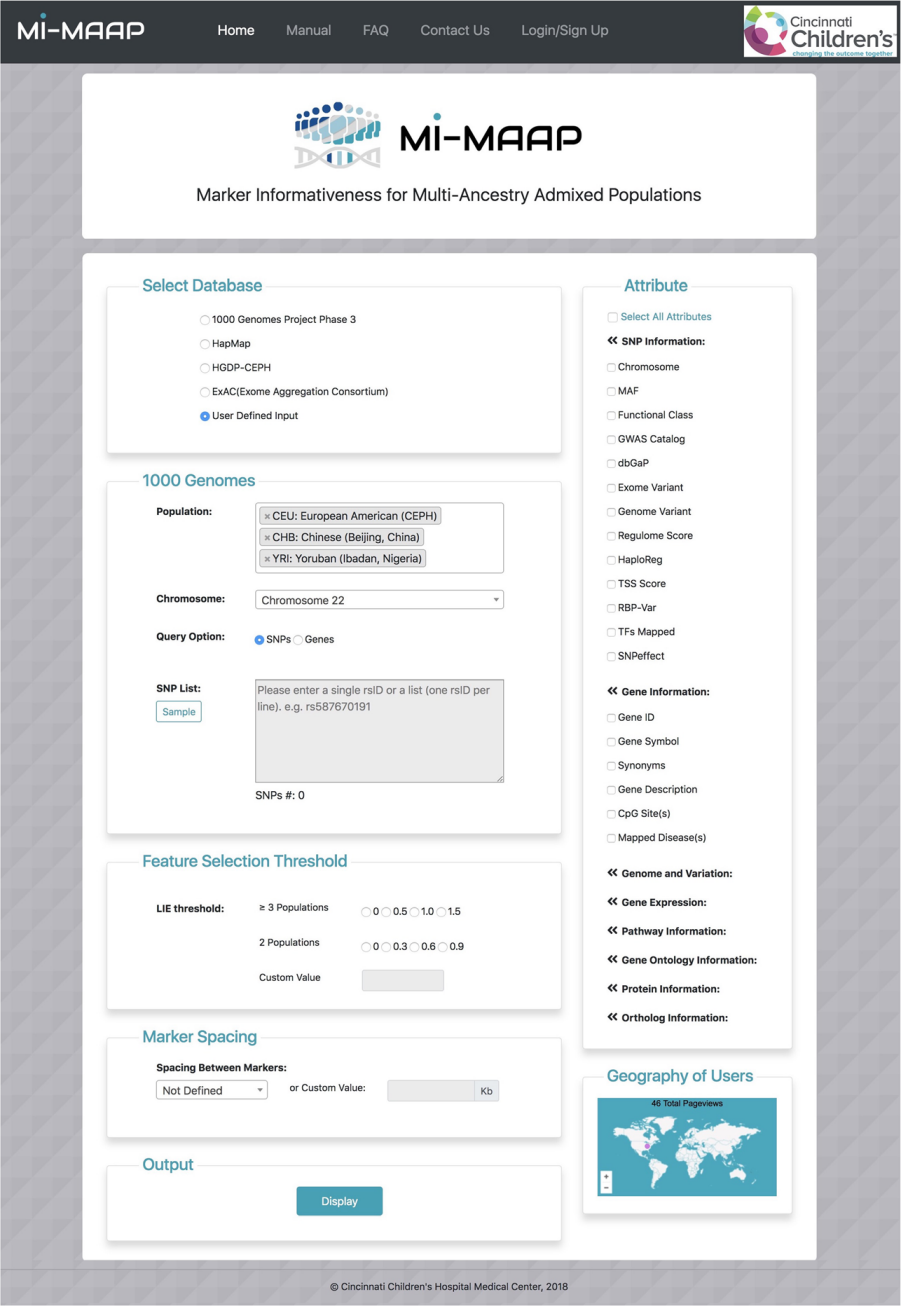
MI-MAAP web interface**.** The architectural design consists of six layers where a user selects populations, markers and filtering criteria to generate AIMs. The web interface allows user to either display the information on the browser or to download to a local hard drive

Figure 3 shows the layout of the MI-MAAP homepage. The overall process of informative SNPs selection from public data is performed in six stages, each building on the results from the preceding stage. The first stage is to select the marker data source. One of the four public databases (1000 Genomes Project, HapMap, HGDP or ExAC) can be selected to retrieve allele frequency data of the SNPs users are interested in. In addition to the public reference datasets, users can upload their own generated data to compute the measure of marker informativeness by selecting the ‘User Defined Input’ radio button. In the second stage, based on the selected database users are able to choose the populations that are included in that database. Meanwhile, users are required to either select a chromosome in the dropdown menu or enter a list of SNPs or a gene name in the given text area. If ‘User Defined Input’ is selected, users need to upload their own SNP allele frequency data or genotype data. A single file in text or excel format of reference data can be uploaded. For the allele frequency data, the reference file must have a header line (SNPID, Chromosome, Ref_Allele, Alt_Allele, Pop1_Freq, …,Popk_Freq) and variants along the rows. Here ‘Popj_Freq’ is the reference allele frequency in population j. In addition, a dynamic formset is attached to enter the number of samples for all the populations used in the data file. For the genotype data, the file must have a header line (SampleID, Population, SNP1, SNP2, …, SNPk) and individual samples along the rows. ‘SNPj’ is the genotype of SNPj in a sample, coded as an integer counting the number of the reference alleles. The third stage is the feature selection threshold section. If the allele frequency data is used, LEI will be computed. If user-generated genotype data is uploaded, users can select one of the methods from LEI, PCA, SVM and RF. LEI values are in the range of 0 to 2 for three or more -population analysis and 0 to 1 for two-population analysis. To set thresholds for LEI and other genotype-data based feature selection approaches (PCA, SVM and RF), users can click on the radio buttons or enter custom values manually in the provided space. The fourth stage is the marker spacing section where the input SNPs list can be further refined by specifying the desired physical distance between markers. Users can select a distance ranging from 50 kb to 5 Mb from the drop-down field or enter a custom value in the unit of kb (1000 base pairs). The fifth stage is the attribute section in the right column. Users can optionally select several attributes through the collapsible menu after entering the necessary input data. In the last stage, by clicking the display button at the bottom of the page, the selected feature selection method will be performed on the given input data and a computation output page will be generated.

### Downloads

The output page contains a table which includes the variant ID, chromosome, reference allele, allele frequencies among the used populations, and the computation values of the selected feature selection method. The output table can be ordered by any columns by clicking the sorting arrow in the column header. The table can also be downloaded to Excel, CSV or PDF files, copied to the clipboard for further use or printed directly. At the bottom of the result page, the previously selected attributes are displayed under each category. Clicking an SNP ID out of the result table redirects to a new page on which the selected attribute information is displayed in a tabulated format. These attribute data are obtained by either directly querying a local API, or providing the hyperlinks to external resources which are shown as the corresponding database logos. For detailed information, we created a fully dedicated website with navigation bar that contains links for manual, frequently asked questions, and contact information and can be accessed at https://research.cchmc.org/mershalab/MI-MAAP/login/. In addition, a detailed documentation including instructions and how to use the tool is provided in Additional file 2: Table S2.

### Genome wide scale and computation time and comparison with other methods

The computation time depends on the number of markers and the feature selection threshold as well as the number of populations that are selected. Mining the information for large chromosomes (e.g. chromosome 1 from 1000 Genome Project - about 6.5 million SNPs) takes longer than mining the information for smaller chromosomes (e.g. chromosome 22 from 1000 Genome Project - about 1.1 million SNPs). For example, with threshold LEI > = 0.2, analyzing LEI values of SNPs in chromosome 1 for CEU, CHB and YRI takes about 32 s and gives 233,636 AIMs, while analyzing SNPs in chromosome 22 is about 6 s and gives 43,532 AIMs. With threshold LEI > = 0.8, analyzing SNPs in chromosome 1 and chromosome 22 for the same populations give an output of 1089 AIMs and 243 AIMs, which take about 26 s and 4 s, respectively. Other genotype-based methods are not scalable to a genome-wide data and cannot be directly compared.

## Illustrative examples

### Ranking ancestry informative markers

**Fig. 4:**
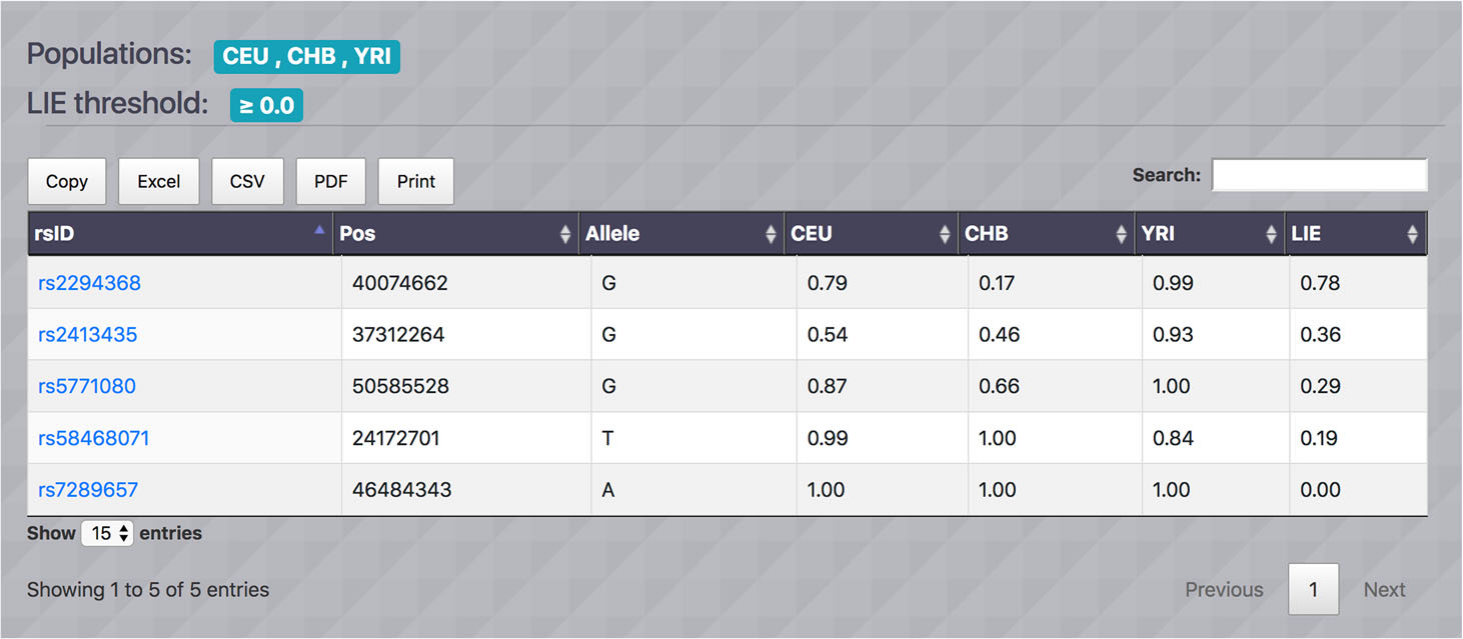
SNPs output display from chromosome 22 for population CEU, CHB and YRI. We have identified a set SNPs on chromosome 22 which we want to investigate the ancestry informativeness of the SNPs among three populations CEU, CHB and YRI. Using MI-MAAP, we compute LEI measure for the desired set of SNPs. The SNP rs2294368 has the highest LEI value (0.78) as it exhibits relatively large allele frequency differences among the three populations (CEU: 0.79, CHB: 0.17, and YRI: 0.99), which suggests that it is the most informative SNP among the SNPs under consideration to be ancestry informative

**Fig. 5:**
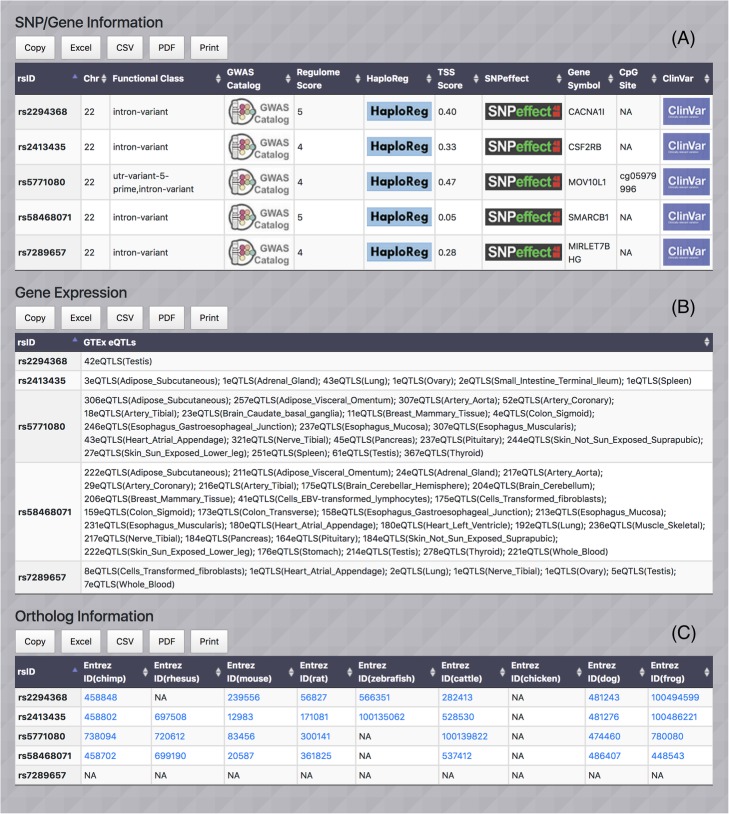
SNP attributes from chromosome 22 for population CEU, CHB and YRI populations: (**a**) SNP/Gene Information, (**b**) Gene Expression Information and (**c**) Ortholog Information

Suppose we have identified a set SNPs on chromosome 22 which we want to investigate the ancestry informativeness of the SNPs among three populations CEU (Northern and Western European), CHB (Han Chinese in Beijing, China) and YRI (Yoruba in Ibadan, Nigeria). Using MI-MAAP tool, we compute LEI measure for the desired set of SNPs. Since all SNPs are investigated, we set threshold LEI > = 0. The corresponding output table with a threshold LEI > = 0 is shown in Fig. 4. SNP rs7289657 has LEI = 0 which implies the SNP is non-informative of the ancestry as expected since the allele frequencies of the three populations are the same. Meanwhile, the SNP rs2294368 has the highest LEI value (0.78) because the SNP exhibits relatively large allele frequency differences among the three populations (CEU: 0.79, CHB: 0.17, and YRI: 0.99), which suggests that it is the most informative SNP among the SNPs under consideration. We further explore the candidate SNP and gene using functional attributes are shown in Fig. 5. The functional annotation scores from RegulomeDB and GWAVA TSS show the SNP rs2294368 has Regulome score = 5 and TSS score = 0.4. By clicking the respective logos, users can get additional association study results from GWAS Catalog and dbGap, and SNP annotation information from HaploReg and SNPeffect. The SNP rs2294368 is located in the Calcium Voltage-Gated Channel Subunit Alpha1 I (CACNA1I) gene. Genome annotation and genomic variation information from ENCODE and ClinVar can be accessed through the hyperlinks provided in the Genome and Variation table. The Gene Expression table displays the number of expression quantitative locus (eQTLs) in different tissues and blood eQTLs for each gene associated with the given SNP. It shows that gene CACNA1I has 42 testis-specific eQTLs and no blood eQTLs. Pathway information is also available by visiting multiple pathway databases KEGG, Reactome and BioCarta. For gene ontology, three main categories, cellular components, biological processes and molecular functions are provided. Gene-associated protein information from four external resources UniProt, Protein Atlas, PFAM and SMART is available. Finally, the Gene Ortholog table contains Entrez Gene IDs for 9 different species (chimpanzee, rhesus, mouse, rat, zebrafish, cattle, chicken, dog and frog) and hyperlinks to the corresponding gene data are provided [18].

### Ancestry classification based on 100 AIMs

**Fig. 6:**
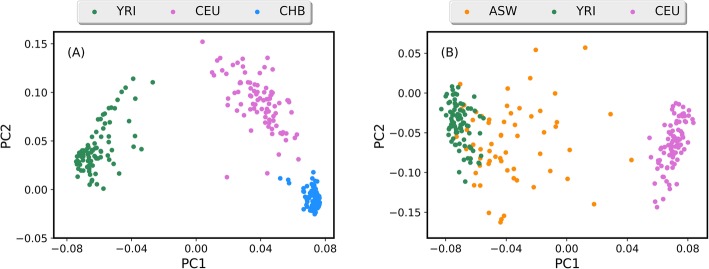
Scatterplots of principal components axis of PC1 and PC2. (**a**) CEU, CHB and YRI populations: the two-dimensional PCA plot reveals distinct separation of CEU, CHB and YRI racial ancestry populations. The first PC contributes 73.5% of the total variation, which clearly distinguishes between Africans from Yoruba and non-Africans samples from Han Chinese. The second PC, contributing 6.8% of the total variation, distinguishes between Europeans and Han Chinese; (**b**) ASW, CEU and YRI populations: CEU and YRI samples form relatively dense clusters, whereas ASW has a lower density and the sample variance is large. Most of the ASW samples are much closer to YRI than CEU and CEU is separated from the other two populations along the PC1 axis. The first PC explains 64.5% of the total variance and the second PC explains 6.4% of the total variance

To demonstrate how MI-MAAP can efficiently extract few AIMs from multiple ancestral populations, and correctly cluster different individuals into their respective geographic populations, the top 100 AIMs selected from 1000 Genome Project dataset were generated using LEI. The genotype data of the top 100 AIMs was examined using the principal components analysis (PCA) algorithm to capture the genetic structure. The PCA plots of the top 100 AIMs with the first and second principal components (PCs) for three continental ancestral populations, CEU (Northern and Western European), CHB (Han Chinese in Beijing, China) and YRI (Yoruba in Ibadan, Nigeria) is shown in Fig. 6a. The two-dimensional PCA plot reveals distinct separation of CEU, CHB and YRI racial ancestry populations. The first PC contributes 73.5% of the total variation, which clearly distinguishes between Africans from Yoruba and non-Africans samples from Han Chinese. The second PC, contributing 6.8% of the total variation, distinguishes between Europeans and Han Chinese. Figure 6b shows the PCA plot using the first and second PCAs for the admixed population ASW (African American) and its continental ancestries CEU and YRI. The first PC explains 64.5% of the total variance and the second PC explains 6.4% of the total variance. CEU and YRI samples form relatively dense clusters, whereas ASW has a lower density and the sample variance is large. Most of the ASW samples are much closer to YRI than CEU and CEU is separated from the other two populations along the PC1 axis. These observations confirm that the African American population is an admixed population with a large contribution from YRI population, and in fact, admixture analyses of ASW samples suggested an average of 80% African ancestry and 20% European ancestry [20–22]. We showed that continental regions can be readily distinguished, while more markers are necessary to improve the classification of closely related and admixed populations. These results further validated that the markers selected from LEI were ancestry informative markers.

### Selection of LEI threshold

To provide more insight into the variation of LEI values in different sets of populations, we analyzed the distribution of LEI in multiple population groups. It should be pointed out that the value of LEI is dependent on multiple factors including the number and genetic diversity among the ancestry populations, level of structures one wishes to correct and the sample size. LEI values are in the range of 0 to 1 for two populations and 0 to 2 for three or more populations. Moreover, LEI values from genetically diverse populations tend to be large while the values from similar populations tend to be smaller. When selecting the LEI threshold for AIMs, it is necessary to consider all these factors together and find the optimal LEI value can achieve the desired performance under different scenarios such as classification accuracy or admixture proportion. As a case study of LEI distribution in different sets of populations, we analyzed the distribution of LEI values and PCA clustering for two population groups, CEU, CHB and YRI, and FIN, GBR and TSI. The maximum LEI score for the diverse ancestral populations CEU-YRI-CHB is 1.21 and for the genetically similar populations FIN-GBR-TSI, the maximum LEI score is 0.52. Additional file 3: Figure S1(A) shows the distribution of LEI values from CEU-YRI-CHB populations for SNPs with LEI > 0.75. In total, we identified 32,530 SNPs with LEI > 0.75 from all autosomal chromosomes and over 2000 SNPs with LEI > 1.0. For the FIN-GBR-TSI populations, we identified 2313 SNPs with LEI > 0.15 and only 251 SNPs with LEI ≥0.25 (Additional file 3: Figure S1(B)). Additional file 4 shows the PCA clustering of the two sets of populations using different LEI thresholds. For CEU-YRI-CHB, SNPs with LEI > 1 separate the three populations as shown in Additional file 4: Figure S2(A). However, for FIN-GBR-TSI, SNPs with LEI ≥0.25 fail to separate three populations and further lowering the LEI threshold to 0.15 improves the PCA clustering (Additional file 4: Figure S2(B-C)). Additionally, only the top 35 LEI SNPs (LEI > 1.0) were adequate to accurately classify the CEU-YRI-CHB populations. A simulation study of the three-way admixed population with CEU, YRI, and CHB as the parental population showed that the top 1000 LEI SNPs (with LEI > 0.833) were required to achieve a root mean square error < 0.05 between the true and estimated ancestry proportion. Because of the variation of the LEI score across different sets of populations, we recommend using an iterative approach to find the optimal set of SNPs that meet the acceptable decision criteria specific to research questions.

## Conclusion

Although a large number of SNPs can provide high-resolution genomic information for ancestry, a small but robust set of SNPs may be more desirable for certain applications including population structure and sample classification based on continental ancestry [23]. The small number of ancestry panel is easy to implement in cost-effective routine laboratories for forensic science and disease genetics studies in resource constraint environment. In this study, we developed MI-MAAP, a web-based tool using an allele frequency-based method to identify smaller subsets of informative markers for multi-ancestry admixed populations as well as retrieving SNP/gene related functional annotation. Building MI-MAAP based on allele frequency data without requiring the individual-level genotype data is appealing in the big data era. In summary, we used a case study to demonstrate that MI-MAAP can explore the information in the genomic data more sufficiently and efficiently from allele frequency data. We believe MI-MAAP is a useful tool for SNP panel developments in population, evolutionary, forensic and disease genetics studies.

## Availability and requirements

Project name: MI-MAAP.

Project home page: https://research.cchmc.org/mershalab/MI-MAAP/login/

Project documentation page: https://research.cchmc.org/mershalab/MI-MAAP/manual/

Operating system(s): Platform independent.

Programming language: Python, JavaScript, HTML, CSS, PHP.

Other requirements: JavaScript enabled web browsers.

License: GNU General Public License.

Any restrictions to use by non-academics: GNU.

## Supplementary information

**Additional file 1: Table S1.** Reference population with labels, sample size that are available from each public database to infer ancestry and web link.

**Additional file 2: Table S2.** Step-by-step manual: Documentation including instructions and how to use the tool in developing ancestry SNP markers in multi-ancestry population.

**Additional file 3: Figure S1.** Distribution of LEI scores. (A) Histogram shows the distribution of LEI scores with threshold 0.75 computed among CEU-YRI-CHB populations. (B) Histogram shows the distribution of LEI scores with threshold 0.15 computed among FIN-GBR-TSI populations.

**Additional file 4: Figure S2.** PCA plots using different LEI thresholds. (A) PC1 vs PC2 produce clear separation of three ancestral populations CEU, YRI, and CHB genome-wide markers with LEI ≥1. (B) PC1 vs PC2 produce some separation of three closely related populations FIN, GBR, and TSI using markers with LEI > 0.15. We have used 2313 markers for the analysis. (C) Using markers with higher thresholds of LEI ≥0.25 failed to separate the three populations. Results were based on all 251 markers with LEI ≥0.25. PLINK 2 was used for the PCA analysis.

## Data Availability

The data supporting this work is publicly available. The 1000 Genomes Project, HapMap Project, Human Genome Diversity Project (HGDP) and Exome Aggregation Consortium (ExAC) data are available publicly from each web link as provided in Additional file 1. The MI-MAAP tool and its full documentation are freely available from https://research.cchmc.org/mershalab/MI-MAAP/login/. An accompanying Additional file 2 describes the input files with examples.

## Abbreviations

MI-MAAP: Marker informativeness for multi-ancestry admixed populations
AIMs: Ancestry informative markers
GWAS: Genome wide association studies
GTEX: Genotype-Tissue Expression
ENCODE: The Encyclopedia of DNA Elements
LEI: Lancaster Estimator of Independence
PCA: Principal component analysis
SVM: Support vector machine
RF: Random forest
HGDP: Human genome diversity project
ExAC: Exome aggregation consortium
HWE: Hardy-Weinberg Equilibrium
HapMap: Haplotype Map
LD: Linkage disequilibrium
dbGap: Database of Genotypes and Phenotypes
