# Supplementary material for: MI-MAAP: marker informativeness for multi-ancestry admixed populations
Source: BMC Bioinformatics. 2020 Apr 3;21:131. doi: 10.1186/s12859-020-3462-5 (PMC7119171; doi:10.1186/s12859-020-3462-5)
Supplement: Supplementary file 1 — Additional file 1: Table S1. Reference population with labels, sample size that are available from each public database to infer ancestry and web link. [file 12859_2020_3462_MOESM1_ESM.docx]

|  |
| --- |

**Additional File 1. Reference populations with labels, sample size and web link**

| **Population** | | **Samples** | **Source/Web Link** |
| --- | --- | --- | --- |
| **1000 Genomes Project** | |  | http://ftp.1000genomes.ebi.ac.uk/vol1/ftp/release/20130502/integrated_call_samples_v3.20130502.ALL.panel |
| African American in Southwest USA [ASW] | | 61 |  |
| African Caribbean in Barbados [ACB] | | 96 |  |
| Bengali in Bangladesh [BEB] | | 86 |  |
| European American (CEPH) [CEU] | | 99 |  |
| British From England and Scotland [GBR] | | 91 |  |
| Chinese Dai in Xishuangbanna, China [CDX] | | 93 |  |
| Colombian in Medellín, Colombia [CLM] | | 94 |  |
| Esan in Nigeria [ESN] | | 99 |  |
| Finnish in Finland [FIN] | | 99 |  |
| Gambian in Western Division – Mandinka [GWD] | | 113 |  |
| Gujarati Indians in Houston, Texas, USA [GIH] | | 103 |  |
| Han Chinese in Beijing, China [CHB] | | 103 |  |
| Southern Han Chinese [CHS] | | 105 |  |
| Iberian Populations in Spain [IBS] | | 107 |  |
| Indian Telugu in the U.K. [ITU] | | 102 |  |
| Japanese in Tokyo, Japan [JPT] | | 104 |  |
| Kinh in Ho Chi Minh City, Vietnam [KHV] | | 99 |  |
| Luhya in Webuye, Kenya [LWK] | | 99 |  |
| Mende in Sierra Leone [MSL] | | 85 |  |
| Mexican American in Los Angeles CA USA [MXL] | | 64 |  |
| Peruvian in Lima Peru [PEL] | | 85 |  |
| Puerto Rican in Puerto Rico [PUR] | | 104 |  |
| Punjabi in Lahore, Pakistan [PJL] | | 96 |  |
| Sri Lankan Tamil in the UK [STU] | | 102 |  |
| Toscani in Italia [TSI] | | 107 |  |
| Yoruba in Ibadan, Nigeria [YRI] | | 108 |  |
|  | |  |  |
| HapMap | |  | <https://www.sanger.ac.uk/resources/downloads/human/hapmap3.html> |
| African ancestry in Southwest USA [ASW] | | 87 |  |
| Utah residents with Northern and Western European ancestry from the CEPH collection [CEU] | | 165 |  |
| Han Chinese in Beijing, China [CHB] | | 137 |  |
| Chinese in Metropolitan Denver, Colorado [CHD] | | 109 |  |
| Gujarati Indians in Houston, Texas [GIH] | | 101 |  |
| Japanese in Tokyo, Japan [JPT] | | 113 |  |
| Luhya in Webuye, Kenya [LWK] | | 110 |  |
| Mexican ancestry in Los Angeles, California [MXL] | | 86 |  |
| Maasai in Kinyawa, Kenya [MKK] | | 184 |  |
| Toscani in Italia [TSI] | | 102 |  |
| Yoruba in Ibadan, Nigeria [YRI] | | 203 |  |
|  | |  |  |
| ExAC(Exome Aggregation Consortium) | |  | http://exac.broadinstitute.org/faq |
| African / African American [AFR] | | 5203 |  |
| American [AMR] | | 5789 |  |
| East Asian [EAS] | | 4327 |  |
| Finnish [FIN] | | 3307 |  |
| Non-Finnish European [NFE] | | 33370 |  |
| South Asian [SAS] | | 8256 |  |
|  | |  |  |
| HGDP | |  | http://www.cephb.fr/hgdp/main.php |
| Africa | BantuKenya | 12 |  |
|  | BantuSouthAfrica | 8 |  |
|  | BiakaPygmy | 36 |  |
|  | Mandenka | 24 |  |
|  | MbutiPygmy | 15 |  |
|  | San | 7 |  |
|  | Yoruba | 25 |  |
| America | Colombian | 13 |  |
|  | Karitiana | 24 |  |
|  | Maya | 25 |  |
|  | Pima | 25 |  |
|  | Surui | 21 |  |
| Central South Asia | Balochi | 25 |  |
|  | Brahui | 25 |  |
|  | Burusho | 25 |  |
|  | Hazara | 25 |  |
|  | Kalash | 25 |  |
|  | Makrani | 25 |  |
|  | Pathan | 25 |  |
|  | Sindhi | 25 |  |
|  | Uygur | 10 |  |
| East Asia | Cambodian | 11 |  |
|  | Dai | 10 |  |
|  | Daur | 10 |  |
|  | Han | 45 |  |
|  | Hezhen | 10 |  |
|  | Japanese | 31 |  |
|  | Lahu | 10 |  |
|  | Miao | 10 |  |
|  | Mongola | 10 |  |
|  | Naxi | 10 |  |
|  | Oroqen | 10 |  |
|  | She | 10 |  |
|  | Tu | 10 |  |
|  | Tujia | 10 |  |
|  | Xibo | 9 |  |
|  | Yakut | 25 |  |
|  | Yi | 10 |  |
| Europe | Adygei | 17 |  |
|  | Basque | 24 |  |
|  | French | 29 |  |
|  | Italian | 14 |  |
|  | Orcadian | 16 |  |
|  | Russian | 25 |  |
|  | Sardinian | 28 |  |
|  | Tuscan | 8 |  |
| Middle East | Bedouin | 49 |  |
|  | Druze | 48 |  |
|  | Mozabite | 30 |  |
|  | Palestinian | 51 |  |
| Oceania | Melanesian | 22 |  |
|  | Papuan | 17 |  |
