# Supplementary material for: MI-MAAP: marker informativeness for multi-ancestry admixed populations
Source: BMC Bioinformatics. 2020 Apr 3;21:131. doi: 10.1186/s12859-020-3462-5 (PMC7119171; doi:10.1186/s12859-020-3462-5)
Supplement: Supplementary file 2 — Additional file 2: Table S2. Step-by-step manual: Documentation including instructions and how to use the tool in developing ancestry SNP markers in multi-ancestry population. [file 12859_2020_3462_MOESM2_ESM.pdf]

MI-MAAP User Manual

MI-MAAP is an easy-to-use web-based bioinformatics tool designed for analyzing informative markers for multi-ancestry admixed populations by utilizing feature selection methods and retrieving the associated SNP or gene information from multiple public resources. It integrates a novel allele frequency data based feature selection algorithm, Lancaster Independence Estimator (LIE), as well other genotype data based methods such as PCA, SVM, and Random Forest. LIE is efficient feature selection strategy for determining significant markers from multiple ancestral populations without requiring individual-level genotype data which is usually massive and the computation task can be very expensive. MI-MAAP has a user-friendly interface which provides researchers an easy and fast way to identify and analyze informative ancestry informative markers (AIMs).

Login Page

User has to sign in with their email address in order to access MI-MAAP. First time users will need to register by providing a valid email address and a password.

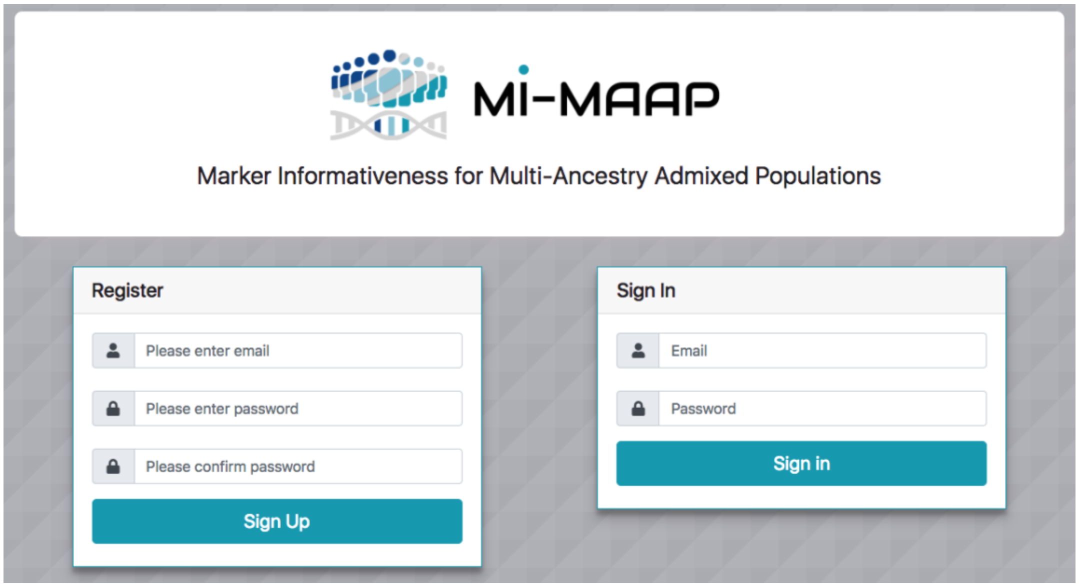

Input Form Page

1. Select Database

1000 Genomes Project Phase III, International Haplotype Map (HapMap), Human Genome Diversity Project (HGDP) and Exome Aggregation Consortium (ExAC) are the databases available with MI-MAAP. Meanwhile, user defined input allows users to upload their own data files.

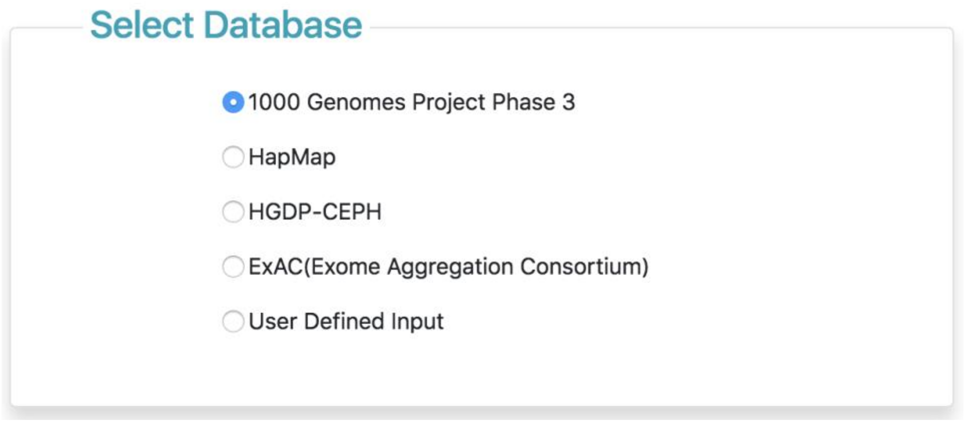

2. Use Public Database

When users use the publicly available genome databases, the next section will ask users to select two or more populations from the selected database.

The population information for the provided four databases are shown below:

1000 Genomes Project: includes 26 populations

Code	Population
**ASW**	African American (Southwest USA)
**BEB**	Bengali (Bangladesh)
**CDX**	Chinese Dai (Xishuangbanna, China)
**CEU**	European American (CEPH)
**CHB**	Chinese (Beijing, China)
**CHS**	Southern Han Chinese
**CLM**	Colombian (Medellin, Colombia)
**ESN**	Esan (Nigeria)
**FIN**	Finnish (Finland)
**GBR**	British (England and Scotland)
**GIH**	Gujarati Indian (Houston, TX)
**GWD**	Gambian (Western Division, The Gambia)
**IBS**	Iberian (Spain)
**ITU**	Indian Telugu (UK)
**JPT**	Japanese (Tokyo, Japan)
**KHV**	Kinh (Ho Chi Minh City, Vietnam)
**LWK**	Luhya (Webuye, Kenya)
**MSL**	Mende (Sierra Leone)
**MXL**	Mexican-American (Los Angeles, California)
**PEL**	Peruvian (Lima, Peru)
**PJL**	Punjabi (Lahore, Pakistan)
**PUR**	Puerto Rican (Puerto Rico)
**STU**	Sri Lankan Tamil (UK)
**TSI**	Toscani (Tuscan, Italy)

**YRI**	Yoruban (Ibadan, Nigeria)

HapMap: includes 11 different populations

Code	Population
**ASW**	African ancestry in Southwest USA
**CEU**	Utah residents with Northern and Western European ancestry from the CEPH collection
**CHB**	Han Chinese in Beijing, China
**CHD**	Chinese in Metropolitan Denver, Colorado
**GIH**	Gujarati Indians in Houston, Texas
**JPT**	Japanese in Tokyo, Japan
**LWK**	Luhya in Webuye, Kenya
**MXL**	Mexican ancestry in Los Angeles, California
**MKK**	Maasai in Kinyawa, Kenya
**TSI**	Toscani in Italia
**YRI**	Yoruba in Ibadan, Nigeria

HGDP: includes 53 populations from 7 different regions

Region	Population
**Africa**	BantuKenya, BantuSouthAfrica, BiakaPygmy, Mandenka, MbutiPygmy, San, Yoruba
**America**	Colomnian, Karitiana, Maya, Pima, Surui
**Central South Asia**	Balochi, Brahui, Burusho, Hazara, Kalash, Makrani, Pathan, Sindhi, Uygur
**East Asia**	Cambodian, Dao, Daur, Han, Han-NChina, Hezhen, Japanese, Lahu, Miao, Mongola, Naxi, Oroqen, She, Tu, Tujia, Xibo, Yakut, Yi
**Europe**	Adygei, Basque, French, Italian, Orcadian, Russian, Sardinian, Tuscan
**Middle East**	Bedouin, Druze, Mozabite, Palestinian
**Oceania**	Melanesian, Papuan

ExAC: includes 6 different populations

Code	Population
**AFR**	African / African American
**AMR**	American
**EAS**	East Asian
**FIN**	Finnish
**NFE**	Non-Finnish European
**SAS**	South Asian

To input markers, users can choose a chromosome number to retrieve all SNPs belonging to that chromosome. In the following text area, users can also type in or copy-paste a list of SNP IDs (one SNP per row) or a single gene name.

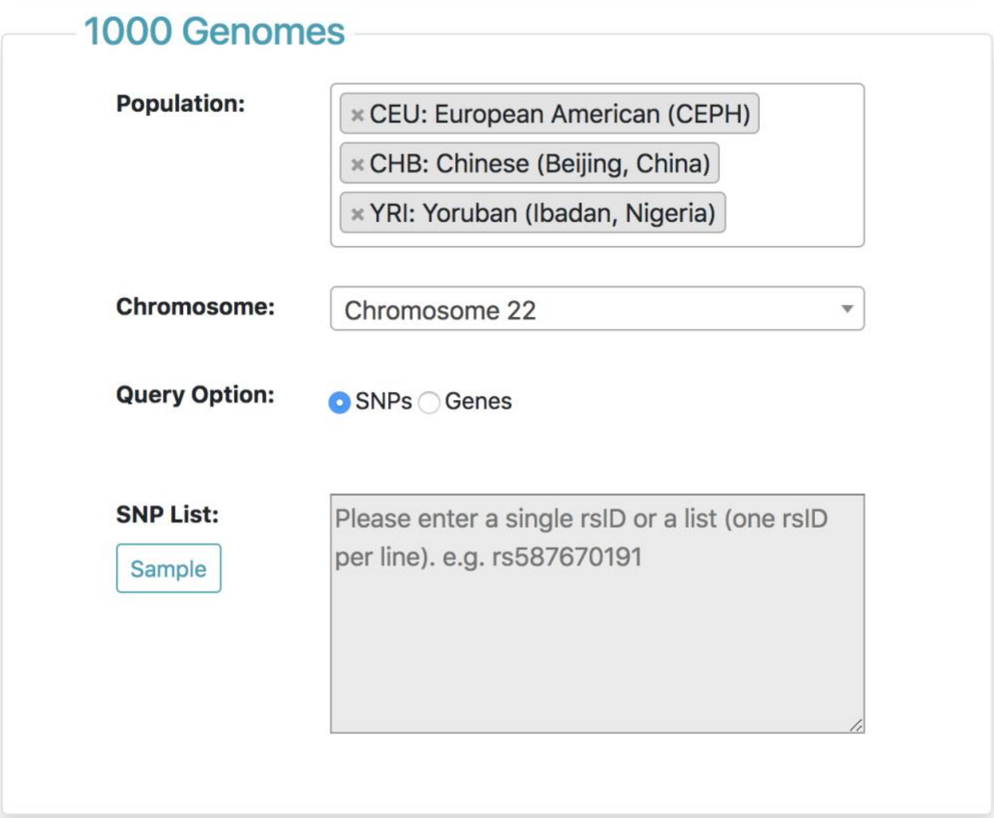

3. **Use User Generated Input**

   When users choose to upload their own data files, a section called "User Defined Input" will show up below the Database Selection. In this section, users are allowed to upload their own allele frequency data or individual-level genotype data. The upload field accepts .txt (tab delimited), .csv, or .xlsx files as input.

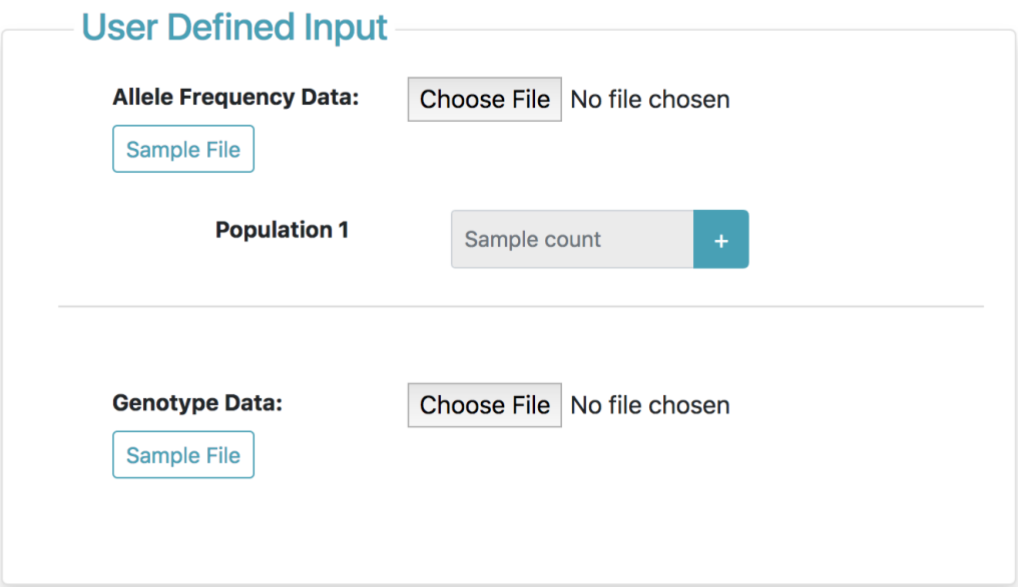

When the allele frequency data is uploaded, users need to enter the number of samples for all the populations used in the data file in the attached dynamic formset, which should have the same population order as in the data file. After clicking display button, LIE values will be computed for each marker in the file. When the genotype data is provided, PCA, SVM and Random Forest will be used to analyze the input variants. The sample files of the allele frequency data and the genotype data are shown below:

SNPID	Chromosome	Ref_Allele	Alt_Allele	Pop1_Freq	Pop2_Freq	Pop3_Freq
rs10158543	1	G	A	0.96	1	0.72
rs10465682	1	A	G	1	1	0.77
rs10749830	1	C	A	0.82	0.57	0.72
rs10799362	1	T	C	0.73	0.72	0.51
rs10907178	1	A	C	0.86	0.85	0.76
rs11121745	1	A	C	0.88	0.88	0.99
rs111569283	1	T	C	0.93	1	1

Sample file of the allele frequency data

sampleID	Population	SNP1	SNP2	SNP3	SNP4	SNP5	SNP6	
sample1	Pop1	1	0	0	0	2	2	
sample2	Pop2	1	2	0	1	2	0	
sample3	Pop2	0	0	1	1	1	0	
sample4	Pop1		1	1	2	1	2	0
sample5	Pop3		2	1	1	0	1	0
sample6	Pop3	1	2	0	2	0	1	
sample7	Pop1		0	0	0	0	0	1
sample8	Pop2	1	2	0	2	1	2	

Sample file of the genotype data

4. Select Threshold for the Feature Selection Method (optional)

User can use either the provided threshold value by selecting the radio button or enter a custom value. When three or more populations are involved, LIE values are in the range of 0 to 2; when only two populations are involved, LIE values are in the range of 0 to 1. A marker with LIE value = 0 carries no information.

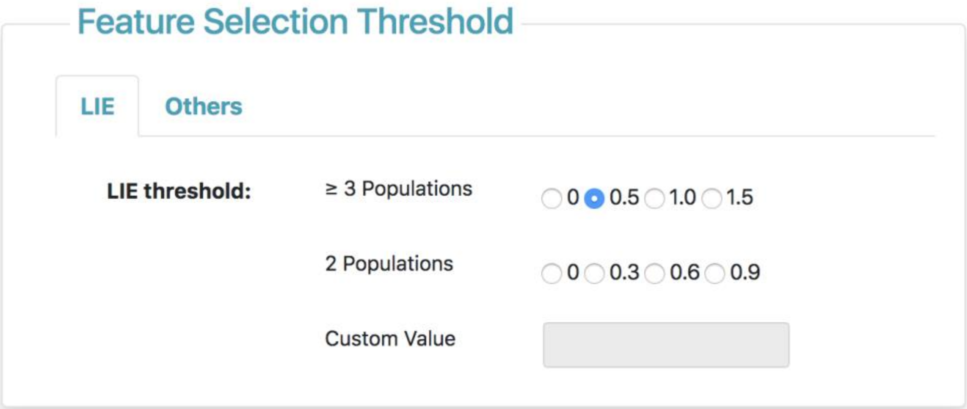

When user-generated genotype data is uploaded, users can also select thresholds for PCA, SVM and Random Forest algorithms by clicking the ‘Others’ tab.

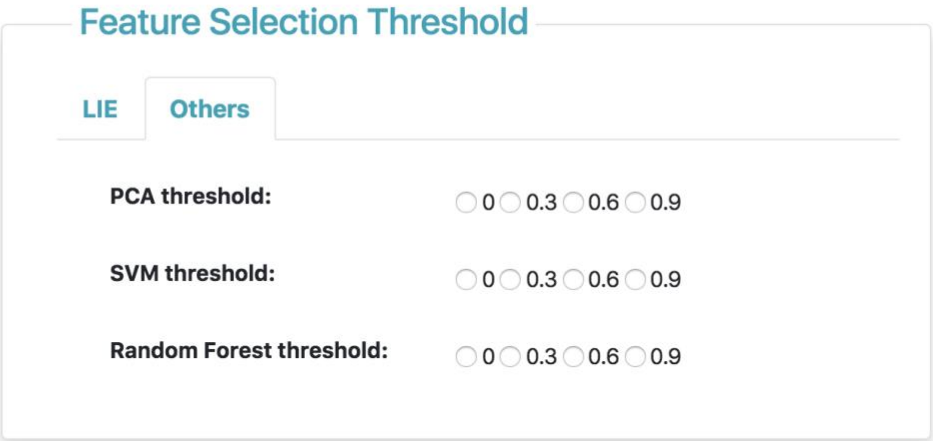

5. **Select Spacing Between Markers (optional)**

Additionally, user can set a value to specify the physical distance between markers by selecting the values provided in the dropdown menu or by entering a custom value in the unit of kb (1000 base pairs).

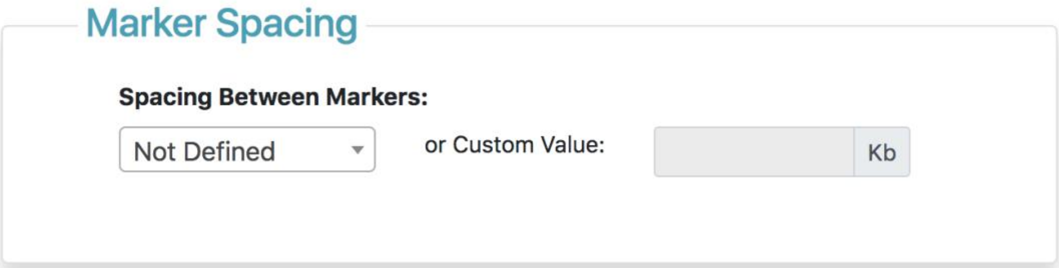

6. **Attributes (optional)**

MI-MAAP also provide users with an option to select different attributes related to the target SNPs.

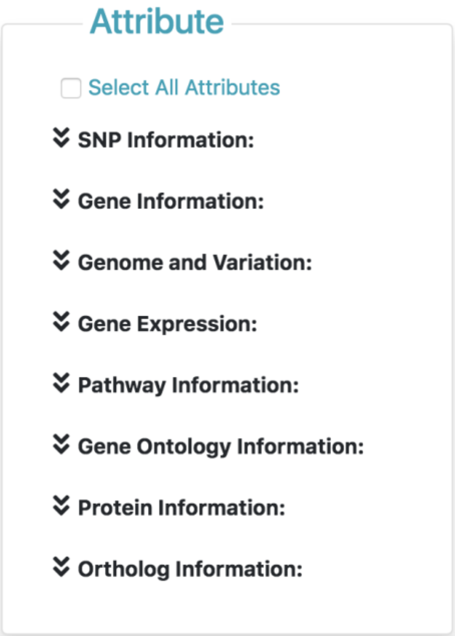

These available attributes are grouped into eight categories: SNP information (chromosome, alleles, MAF, functional class, Regulome Score, TSS Score, and links to GWAS Catalog, dbGap, Exome variant, Genome variant and so on), gene information (such as gene ID, gene symbol, synonyms, gene description, CpG sites and mapped diseases), genome and variation (links to ENCODE, dbVar, ClinVar and BioGPS), gene expression (such as GEO profiles, GTEx eQTL, Blood eQTL and so on), biological pathways (links to KEGG pathways, Reactome and BioCarta), gene ontology (Cellular components, Biological process and Molecular function), protein (links to UniProt, Protein Atlas, PFAM and SMART), and species orthologs (such as Entrez IDs for chimp, rhesus, mouse, rat, zebrafish, cattle, chicken, dog and frog).

By clicking "Select All Attributes" checkbox, all attributes will be selected automatically.

7. **Output**

By clicking the "Display" button, all the input information will be submitted. Once the analysis is finished, a result table will be generated in the output page.

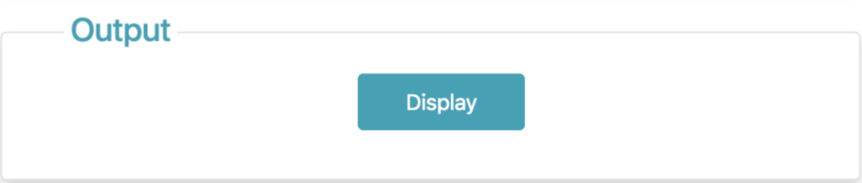

Output Page

Using 1000 Genomes Project database, a sample output of 5 SNPs on chromosome 22 for three populations CEU, CHB and YRI with LIE threshold ≥ 0 is shown in the table below.

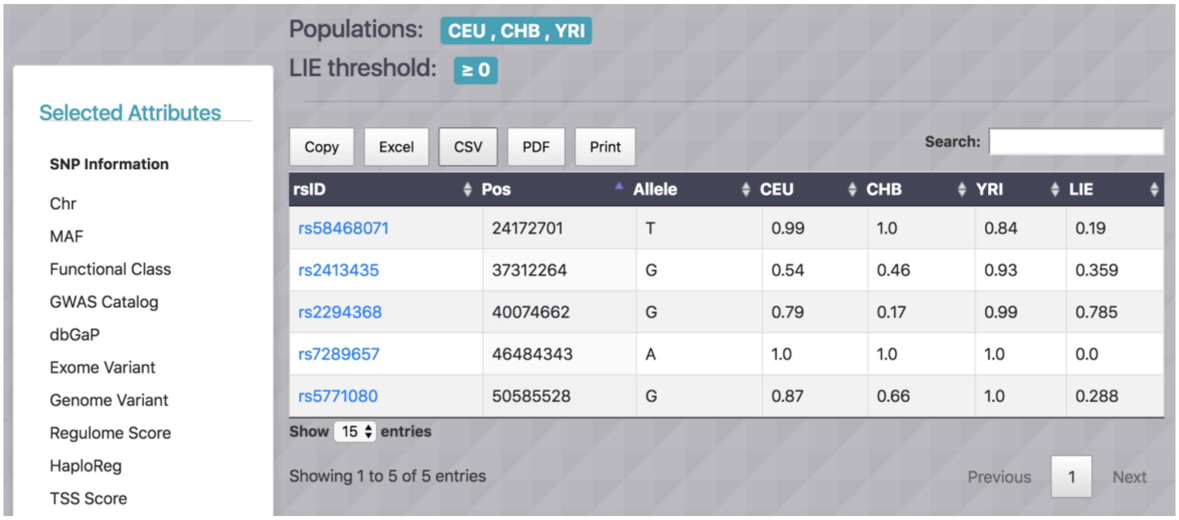

rsID	Pos	Allele	CEU	CHB	YRI	LIE
rs58468071	24172701	T	0.99	1.0	0.84	0.19
rs2413435	37312264	G	0.54	0.46	0.93	0.359
rs2294368	40074662	G	0.79	0.17	0.99	0.785
rs7289657	46484343	A	1.0	1.0	1.0	0.0
rs5771080	50585528	G	0.87	0.66	1.0	0.288

On the top of the table, five buttons are provided for users to access the output: the table can be copied using "Copy" button, downloaded using "Excel", "CSV" or "PDF" buttons, and printed using "Print" button. Number of rows displayed in the table can be adjusted using "Show 15 entries" dropdown menu.

The left column shows all the attributes that users selected in the input form page. To view the attribute information for a SNP, users can click the rsID (in blue) in the output table.

SNP Attribute Page

As an example, the attribute information page for rs58468071 is shown as below. Links to external databases like GWAS Catalog, dbGaP, HaploReg, SNPeffect, BioGPS etc. are embedded into the output table. Likewise, users can save the attribute table by using the five buttons provided on top of each table.

Attribute Information for rs58468071

SNP Information

Copy Excel CSV PDF Print

Attribute	Value
Chr	22
dbGaP	PheGenI Phenotype-Genotype Integrator
Exome Variant	
Functional Class	intron-variant
Genome Variant	
GWAS Catalog	GWAS Catalog
HaploReg	HaploReg
MAF	C(0.0447284)
RBP-Var	NA
Regulome Score	5
SNPeffect	SNPeffect PHENOTYPING HUMAN MUTATIONS
TFs Mapped	NA
TSS Score	0.05

Gene Information

Copy Excel CSV PDF Print

Attribute	Value
CpG Site	NA
Gene Description	SWI/SNF related, matrix associated, actin dependent regulator of chromatin, subfamily b, member 1
Gene ID	6598
Gene Symbol	SMARCB1
Mapped Disease	NA
Synonyms	BAF47,CSS3,INI1,MRD15,PPP1R144,RDT,RTPS1,SNF5,SNF5L1,SWNTS1,Sfh1p,Snr1,hSNFS

Genome and Variation

Copy Excel CSV PDF Print

Attribute	Value
BioGPS	BioGPS.ORG
ClinVar	ClinVar
dbVar	dbVar
ENCODE	ENCODE

Gene Expression

Copy Excel CSV PDF Print

Attribute	Value
Blood eQTLs	102 cis and 0 trans
GEO Profiles	GEO Profiles
GTEx eQTLs	222eQTLS(Adipose_Subcutaneous); 211eQTLS(Adipose_Visceral_Omentum); 24eQTLS(Adrenal_Gland); 217eQTLS(Artery_Aorta); 29eQTLS(Artery_Coronary); 216eQTLS(Artery_Tibial); 175eQTLS(Brain_Cerebellar_Hemisphere); 204eQTLS(Brain_Cerebellum); 206eQTLS(Breast_Mammary_Tissue); 41eQTLS(Cells_EBV-transformed_lymphocytes); 175eQTLS(Cells_Transformed_fibroblasts); 159eQTLS(Colon_Sigmoid); 173eQTLS(Colon_Transverse); 158eQTLS(Esophagus_Gastroesophageal_Junction); 213eQTLS(Esophagus_Mucosa); 231eQTLS(Esophagus_Muscularis); 180eQTLS(Heart_Atrial_Appendage); 180eQTLS(Heart_Left_Ventricle); 192eQTLS(Lung); 236eQTLS(Muscle_Skeletal); 217eQTLS(Nerve_Tibial); 184eQTLS(Pancreas); 164eQTLS(Pituitary); 184eQTLS(Skin_Not_Sun_Exposed_Suprapubic); 222eQTLS(Skin_Sun_Exposed_Lower_leg); 176eQTLS(Stomach); 214eQTLS(Testis); 278eQTLS(Thyroid); 221eQTLS(Whole_Blood)
Promoter(GE)	ENCODE
Transcript ID	ENST00000344921.6

Pathway Information

Copy Excel CSV PDF Print

Attribute	Value
BioCarta	BIOCARTA
KEGG Pathways	KEGG
Reactome	reactome

Gene Ontology Information

Copy Excel CSV PDF Print

Attribute	Value
Biological Process	14
Cellular Component	10
Molecular Function	4

Protein Information

Copy Excel CSV PDF Print

Attribute	Value
PFAM	Pfam
Protein Atlas	THE HUMAN PROTEIN ATLAS
SMART	SMART
UniProt	UniProt

Ortholog Information

Copy Excel CSV PDF Print

Attribute	Value
Entrez ID(cattle)	537412
Entrez ID(chicken)	NA
Entrez ID(chimp)	458702
Entrez ID(dog)	486407
Entrez ID(frog)	448543
Entrez ID(mouse)	20587
Entrez ID(rat)	361825
Entrez ID(rhesus)	699190
Entrez ID(zebrafish)	NA

An Example of Racial Ancestry Classification by Using LIE

To demonstrate how MI-MAAP can efficiently extract ancestral informative markers (AIMs) and cluster different individuals into their geographic populations, the principal components analysis (PCA) algorithm was used to analyze the top 100 AIMs that were generated using LIE from the 1000 Genome Project dataset. The PCA plots for two sets of three populations, CEU, CHB and YRI, and ASW, CEU and YRI are shown below.

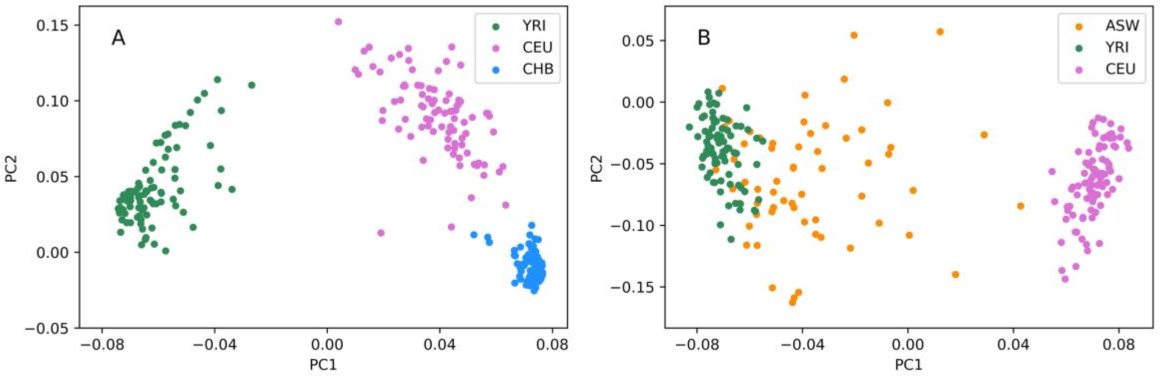

PCA clustering of parental populations: scatterplots of principal components axis one (PC1) and axis two (PC2) for (A) CEU, CHB and YRI populations, and (B) ASW, CEU and YRI populations.

As one can expect, Figure(A) shows a distinct separation of the three continental ancestral populations CEU, CHB and YRI. In Figure(B) CEU and YRI are clearly separated, but ASW shows a lower density with a large sample variance. Meanwhile, most of the ASW samples are much closer to YRI than CEU and CEU is separated from the other two populations along PC1 axis. This is because African American population is an admixed population with an average of 80% African ancestry and 20% European ancestry. We showed that continental regions can be clearly distinguished, while more markers are necessary to improve the classification of closely related and admixed populations. These observations confirm that the markers selected using LIE were ancestry informative markers.
