# Supplementary figures and images for: MI-MAAP: marker informativeness for multi-ancestry admixed populations
Source: BMC Bioinformatics. 2020 Apr 3;21:131. doi: 10.1186/s12859-020-3462-5 (PMC7119171; doi:10.1186/s12859-020-3462-5)

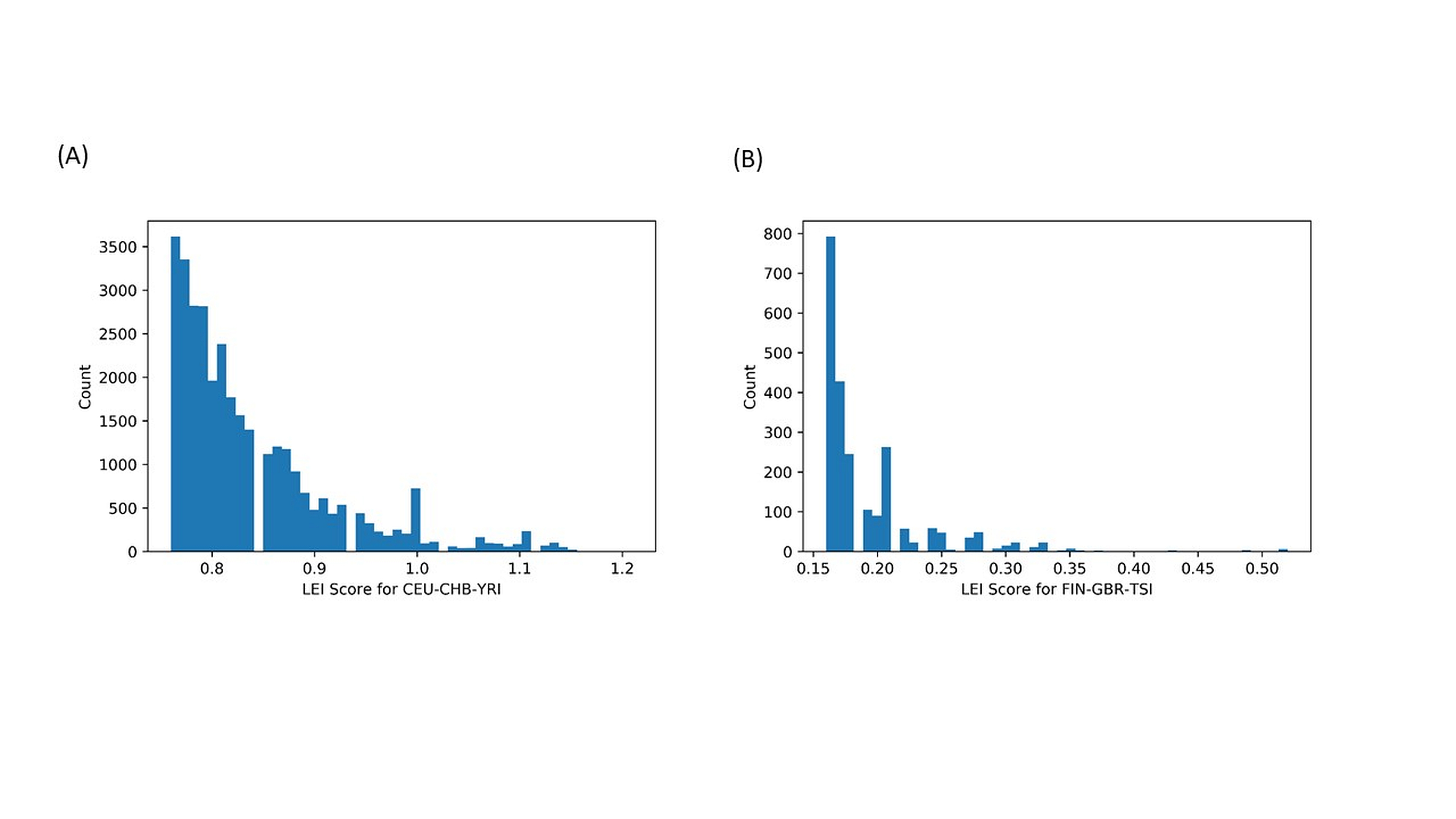

Supplement: Supplementary file 3 — Additional file 3: Figure S1. Distribution of LEI scores. (A) Histogram shows the distribution of LEI scores with threshold 0.75 computed among CEU-YRI-CHB populations. (B) Histogram shows the distribution of LEI scores with threshold 0.15 computed among FIN-GBR-TSI populations. [file 12859_2020_3462_MOESM3_ESM.jpg]

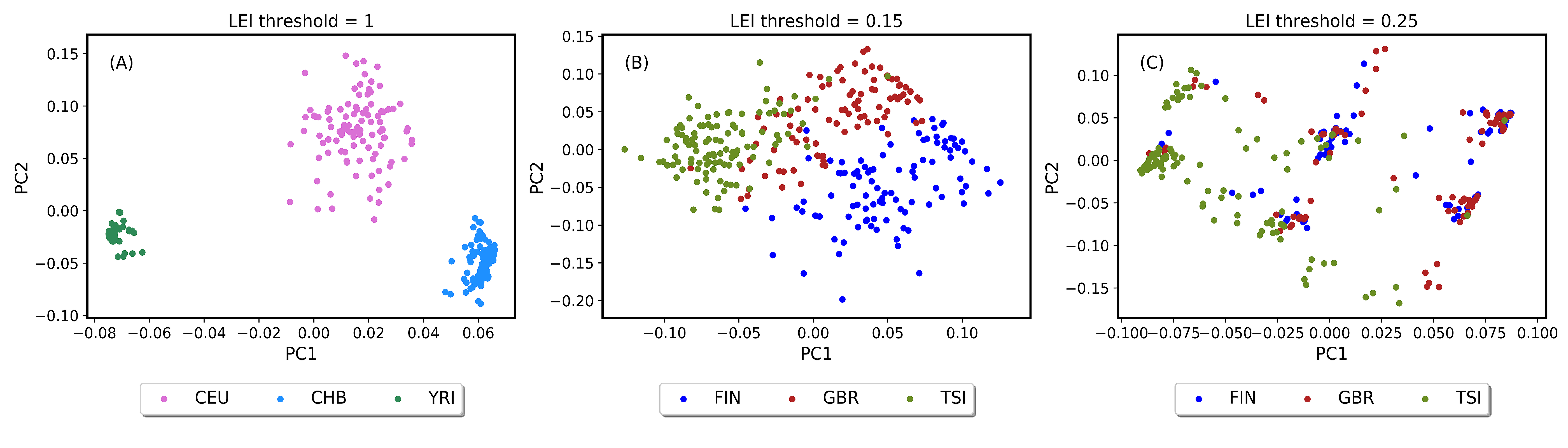

Supplement: Supplementary file 4 — Additional file 4: Figure S2. PCA plots using different LEI thresholds. (A) PC1 vs PC2 produce clear separation of three ancestral populations CEU, YRI, and CHB genome-wide markers with LEI ≥1. (B) PC1 vs PC2 produce some separation of three closely related populations FIN, GBR, and TSI using markers with LEI > 0.15. We have used 2313 markers for the analysis. (C) Using markers with higher thresholds of LEI ≥0.25 failed to separate the three populations. Results were based on all 251 markers with LEI ≥0.25. PLINK 2 was used for the PCA analysis. [file 12859_2020_3462_MOESM4_ESM.jpg]
